# Low temperature catalytic hydrodeoxygenation of lignin-derived phenols to cyclohexanols over the Ru/SBA-15 catalyst†

**DOI:** 10.1039/d2ra01183b

**Published:** 2022-03-24

**Authors:** Shanshan Feng, Xudong Liu, Zhishan Su, Guiying Li, Changwei Hu

**Affiliations:** Key Laboratory of Green Chemistry and Technology, Ministry of Education, College of Chemistry, Sichuan University Chengdu Sichuan 610064 P. R. China changweihu@scu.edu.cn; State Key Laboratory of Utilization of Woody Oil Resource, Hunan Academy of Forestry Sciences Changsha 410004 China

## Abstract

Cyclohexanol and its derivatives are widely used as chemical intermediates and fuel additives. Herein, Ru/SBA-15 catalysts were prepared *via* impregnation, and used for the production of cyclohexanols from lignin-derived phenols. The catalyst samples were characterized by XRD, XPS, TEM, *etc.*, where the Ru^0^ species was speculated as the active phase. 5 wt% Ru/SBA-15 with small Ru particle size (4.99 nm) and high Ru dispersion (27.05%) exhibited an excellent hydrogenation activity. A high cyclohexanol yield of >99.9% was achieved at 20 °C for 5 h in an aqueous phase, and the catalyst indicated stable activity and selectivity after five runs. Crucially, Ru/SBA-15 exhibited a zero-order reaction rate with an apparent activation energy (Ea) as low as 10.88 kJ mol^−1^ and a TON of 172.84 at 80 °C. Simultaneously, demethoxylation activity was also observed in the hydrodeoxygenation (HDO) of G- and S-type monophenols, and a high yield of 37.4% of cyclohexanol was obtained at 80 °C and 4 h when using eugenol as substrate.

## Abstract 2

Ru/SBA-15 showed excellent activity for phenol to cyclohexanol at 20 °C and exhibited a zero-order character with low Ea of 10.88 kJ mol^−1^. A high yield of 37.4% of cyclohexanol was obtained at 80 °C and 4 h when using eugenol as substrate.

## Graphical abstract

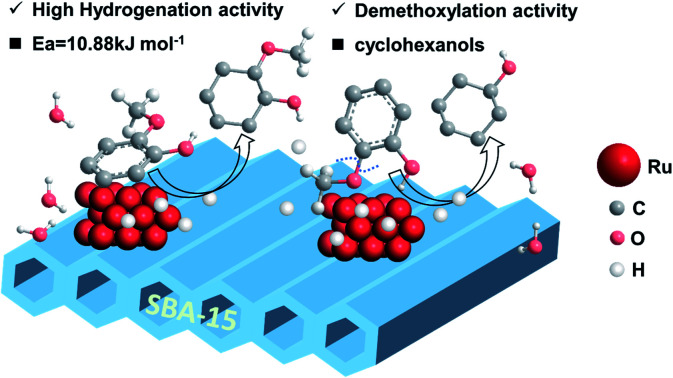

## 1. Introduction

Due to the excessive exploitation and utilization of fossil resources, human society is troubled by resource exhaustion and a series of environmental problems. The valorization of renewable resources has become one of the key fields to reduce the gradual depletion of petroleum reserves and rigorous environmental problems, and lignocellulose biomass is one of the feedstocks with most potential which could be upgraded to high-quality fuels and chemicals.^1–3^ However, lignin has been under-utilized compared to carbohydrates, and often discarded as waste.^4^

The decomposition of lignin typically affords phenols, the hydrodeoxygenation (HDO) of which is extremely challenging.^5–9^ An effective method to address the low economic benefit in biorefinery is the further refining of the monophenols to cyclohexanols and cyclohexanones as well as hydrocarbon compounds.^10^ Besides, this process can valorize the high-value of lignin and alleviate the increasing pressure caused by the depletion of fossil resources and environmental problems.^11,12^ Among which, cyclohexanols were widely used as an intermediate in industrial processes, and phenol hydrogenation and cyclohexane oxidation were proven techniques from the twentieth century to produce cyclohexanols. However, obtaining cyclohexanol from phenol had been eliminated, for phenol was relatively expensive and scarce from fossil resource compared with cyclohexane. Therefore, upgrading of lignin-derived phenols to obtain cyclohexanol and its derivatives will be a promising way from abundant renewable raw materials without side reactions.

Transition metal especially noble metal catalysts (Pd, Pt, Ru, Rh, *etc.*) showed excellent performance in the upgrading of lignin-derived phenols.^10,13^ The product distribution was associated with the adsorption behavior of the catalyst. Generally, the phenol compounds adsorbed on the active metal species by the exchange of the π-electron density of aromatic ring and the d band electron density of metal, and the electronic interactions would result in the decrease of the aromaticity of the reactant, thus result in the activity of hydrogenation and deoxygenation.^14–16^ Moreover, the adsorption mode of phenol on catalyst would affect the selectivity of cyclohexanol or cyclohexanone, and the deoxygenation was usually ascribed to the Brønsted acidity of the catalyst.^3,17–19^ Ru catalysts have consistently presented excellent catalytic activity in the HDO of phenolic compounds (see Table S1 in ESI†). Ishikawa *et al.*^20^ generated a Ru–MnO_*x*_ catalyst and used it to convert guaiacol to cyclohexanols (81%) at 160 °C in water. Similarly, Sreenavya *et al.*^21^ reported the conversion of eugenol in the presence of (NiRu-HT)-type material, and high selectivity to cyclohexanols (83.8%) were achieved at 150 °C and 2.5 MPa H_2_. It was assumed that the formation of metallic Ru species on the surface of catalyst facilitated both the cleavage of the C_aryl_–OCH_3_ bond (demethoxylation) and the hydrogenation of aromatic ring to form cyclohexanols. However, the temperature of the HDO reaction was relatively high and might result in the deactivation of catalyst, where the carbon deposition might occur and block the active sites of both metal and the carrier of the catalyst.^22,23^

Crucially, solvent exhibited an important role in HDO, and water is a very interesting solvent in particular, which could modify the transition state of phenols. It was reported that guaiacol would loss aromaticity and form its keto isomer due to the incipient isomerization in water, and enol and keto isomers coexist even without catalysts.^24^ In addition, water as a polar solvent could promote both hydrogenation and demethoxylation of methoxy-substituted monophenols compared with non-polar solvents as found from density functional theory (DFT) studies of phenol.^25,26^ However, there was relatively less reports for the HDO of lignin-derived monophenols at low temperature (<100 °C) (Table S1†). In the present study, Ru/SBA-15 was prepared for the conversion of phenol and other lignin-derived phenols in water, and the kinetic study of phenol hydrogenation to cyclohexanol was performed under kinetically controlled conditions. Ru/SBA-15 exhibited an excellent activity for the hydrogenation of aromatic ring with a zero-order character. Moreover, the catalyst also showed demethoxylation activity in the conversion of methoxy-substituted monophenols, and the probable pathways of obtaining demethoxylation and/or hydrogenation products were analyzed.

## 2. Experimental

### 2.1 Materials

Phenol (99%, GC), 4-ethylphenol (99%, GC), 4-propylphenol (99%, GC), 4-methylguaiacol (98%, GC), 4-allylguaiacol (99%, GC), syringol (98%, GC) and 4-methylsyringol (98%, GC) were purchased from Tokyo Chemical Industry Co., (TCI). Guaiacol (99%, GC), 4-ethylguaiacol (>98%) were purchased from Adamas. SBA-15 was purchased from XFNANO. All these chemicals obtained from commercial resources were used directly.

### 2.2 Catalyst preparation

Ru/SBA-15 catalysts were prepared using RuCl_3_ (45–55%, Adamas) as a precursor, employing the incipient-wetness impregnation method. SBA-15 carrier was calcined at 550 °C for 4 h, then tablet grinding to 80–120 meshes. Typically, the pre-treated SBA-15 carrier was dropped to the aqueous solution of RuCl_3_ and ultrasonically dispersed for 1 h, then shaking at 30 °C for 24 h. The catalyst precursor was dried for about 2 h in a water bath firstly and then in an oven overnight both at 80 °C, then calcined in muffle furnace at 400 °C (2 °C min^−1^) for 4 h. Before reaction, the Ru/SBA-15 catalyst was pre-reduced by H_2_ at 300 °C for 2 h with a flow rate of 40 mL min^−1^.

### 2.3 Catalyst characterization

Structural and chemical properties of SBA-15 support and Ru/SBA-15 catalysts were characterized by N_2_ adsorption–desorption, transmission electron microscope (TEM), X-ray diffraction (XRD), X-ray photoelectron spectroscopy (XPS), and Inductively Coupled Plasma Atomic Emission Spectrometer (ICP-AES). Additional details and operation parameters are provided in ESI†.

The metal dispersion (*D*) of the samples were calculated by the particle diameter (*d*) of Ru obtained from TEM according to the procedures described by Anderson as the following equation:1
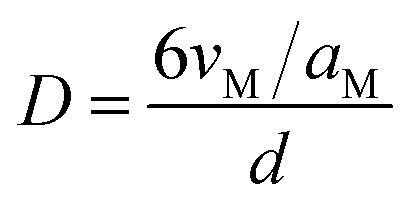
where *v*_M_ and *a*_M_ were the volume per metal atom in the bulk and the effective average area occupied by a ruthenium atom in the surface, respectively. Herein, *v*_M_ = *M*_w_/(*ρ N*_o_) (*M*_w_ is the atomic weight, *ρ* is the density, and *N*_o_ is Avogadro's number), *a*_M_ = 1/(1.63 × 10^19^) (1.63 × 10^19^ is the number of surface atoms per square meter of the polycrystalline ruthenium surface).^27,28^ Hence, using the values of *M*_w_, *ρ*, and *N*_o_,2
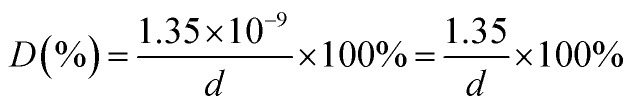


### 2.4 Catalyst test

The hydrogenation of lignin-derived phenols was performed in a 100 mL Parr reactor. The fluid was analyzed qualitatively and quantitatively by GC-MS (Agilent 6820) and GC-FID (PerkinElmer Clarus 580) for yield calculation. Acetophenone was used as internal standard. Additional details and operation parameters are provided in the ESI.†

The conversion (Conv.) of the reactant and the selectivity (Sel.) towards one specific product, as well as the turnover number (TON, in moles reactant per mole of Ru exposed on the surface) were calculated by the following equations:3
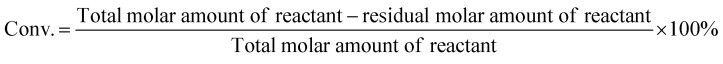
4
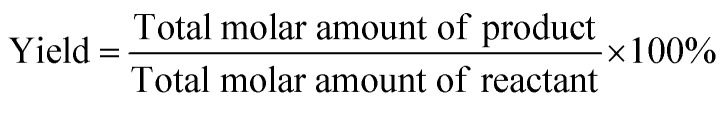
5
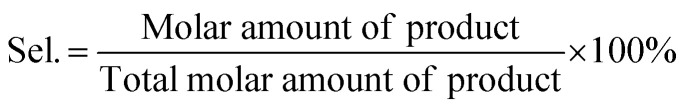
6
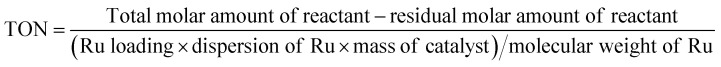


### 2.5 Catalyst recycle

After reaction, the used catalyst was separated from the solution by filtration, washed with ethanol, and then dried in an oven at 80 °C for 1 h. After that, the catalyst was used for the next run without other treatments.

## 3. Results and discussion

### 3.1 Catalyst characterization

**Fig. 1:**
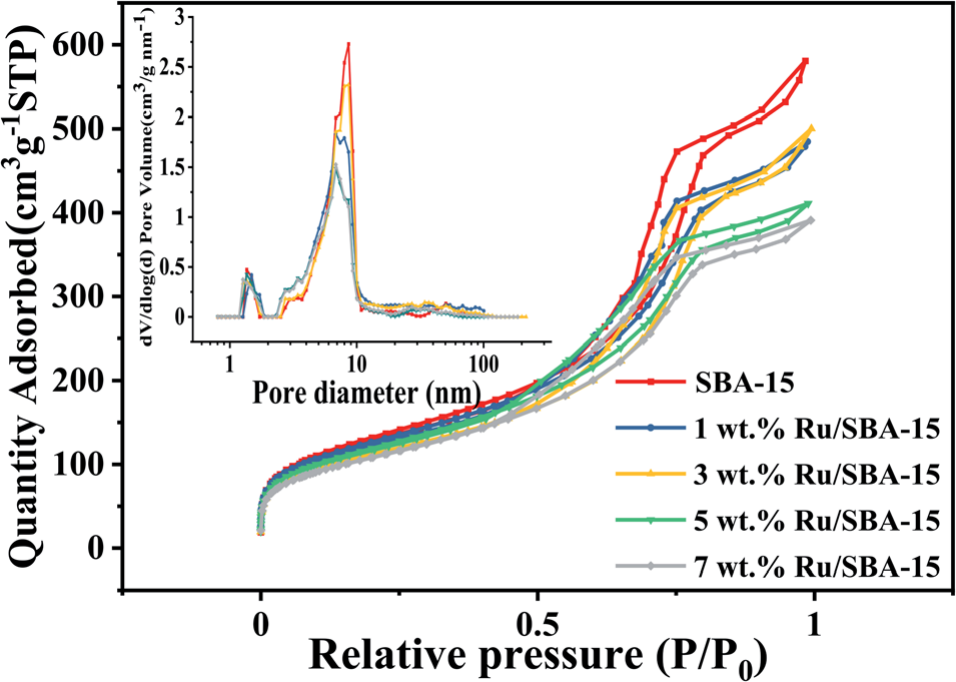
N_2_ adsorption–desorption isotherms the pore size distribution of Ru/SBA-15.

The textural characteristics of the catalysts with different Ru loadings (1–7 wt%) on SBA-15 were summarized in Table 1. The actual metal loading of the catalysts suggested that almost all ruthenium were successfully loaded on SBA-15 carrier for 1–7 wt% Ru loading catalysts. Fig. 1 showed the N_2_ adsorption–desorption isotherms and pore size distribution of Ru/SBA-15 catalysts and SBA-15 carrier. The isotherms all showed a representative IV-type with the hysteresis loops of typical H1-type, implying a uniform pipe diameter material with open ends, which indicated that the skeleton structure of the support was retained without destroy during the preparation of the catalysts.^29^ According to the surface area and pore size, it was indicated that there were partial Ru species loaded on the surface of the support. Crucially, the dispersive Ru particles might also partially enter the inner hole of the carrier and anchored on the wall of the pores, where the pore size ranged from 7 to 9 nm was decreased gradually with increasing Ru loadings from 1 wt% to 7 wt%.

**Table 1:** The textural properties of the Ru/SBA-15 catalysts

Catalyst	*S*_BET_ a (m^2^ g^−1^)	*V*_p_ a (cm^3^ g^−1^)	*D*_p_ a (nm)	Ru loadings^b^ (wt%)	*d*_Ru_ c (nm)	*D*_Ru_ c (%)	TON^d^
SBA-15	481.8	0.86	7.16	—		—	—
1 wt% Ru/SBA-15	459.4	0.74	6.29	0.95	7.19	18.78	467.50
3 wt% Ru/SBA-15	407.6	0.74	6.96	2.81	6.02	22.43	223.76
5 wt% Ru/SBA-15	432.8	0.63	5.72	4.66	4.99	27.05	172.84
7wt% Ru/SBA-15	394.8	0.60	5.52	6.15	6.14	21.99	118.68

a *S*_BET_ represented the surface area determined from the Brunauer-Emmett–Teller (BET) equation, and *V*_p_, and *D*_p_ represented the average pore volume and average pore diameter measured by Barrett–Joyner–Halenda (BJH) formula from N_2_ adsorption–desorption.

b Actual Ru loadings was measured by ICP-AES.

c Ru dispersion (*D*_Ru_) was calculated by the formula: *D*_Ru_ = 1.35/*d*_Ru_ (Ru particle size (*d*_Ru_) obtained from TEM images in Fig. 2).

d TON based on the reactant conversion at 80 °C for 1.5 h and number of active Ru particles exposed on the surface of catalysts (*D*_Ru_).

Fig. 2 showed the XRD patterns of the samples, where the peak intensity increased gradually with Ru loading. Characteristic diffraction peaks of Ru^0^ were observed at 2*θ* = 38.4°, 42.1°, 44.0°, 58.2°, 69.4° and 78.4°, which could be corresponded to hcp Ru (100), (002), (101), (102), (110) and (103) (PDF#06-0663).^30,31^ The Ru particles all distribute uniformly with clear edges in the samples of 1–7 wt% Ru/SBA-15 catalysts as shown in TEM images (Fig. 3). As for 1 wt% and 3 wt% Ru/SBA-15 catalysts, the number of Ru particles was relative less than that of 5 wt% Ru/SBA-15. However, the Ru particles occurred obvious agglomerations and covered the carrier (Fig. 3d) when the Ru loadings reached to 7 wt%, which might explain the results of N_2_ adsorption–desorption. The mean particle diameter of Ru showed a tendency to decrease at first and then increase consistently with the Ru loading, where the minimum particle diameter appears at 5 wt% Ru/SBA-15 catalyst with the mean size of 4.99 nm. The particle diameter calculated by Scherrer formula based on Ru(101) in XRD had the similar tendency with TEM (Table S2†). Herein, the dispersion (*D*) of active Ru particles were calculated by the mean particle diameter as the equation described above (*D* = 1.35/*d*) and the corresponding results were shown in Table 1. Among which, 5 wt% Ru/SBA-15 showed the highest dispersion (27.05%) compared with other samples, which implied a greater quantity of active Ru atoms exposed on the surface of the catalyst and a better ability to adsorb and activate H_2_ molecules.^32^ The lattice spacing of 0.204, 0.213, and 0.233 nm measured in HRTEM images of 5 wt% Ru/SBA-15 (Fig. 3e) could be corresponded to the (101), (002), and (110) planes of hexagonal close packed (hcp) Ru, and it was consistent well with the crystal planes shown in XRD patterns.^32–34^

**Fig. 2:**
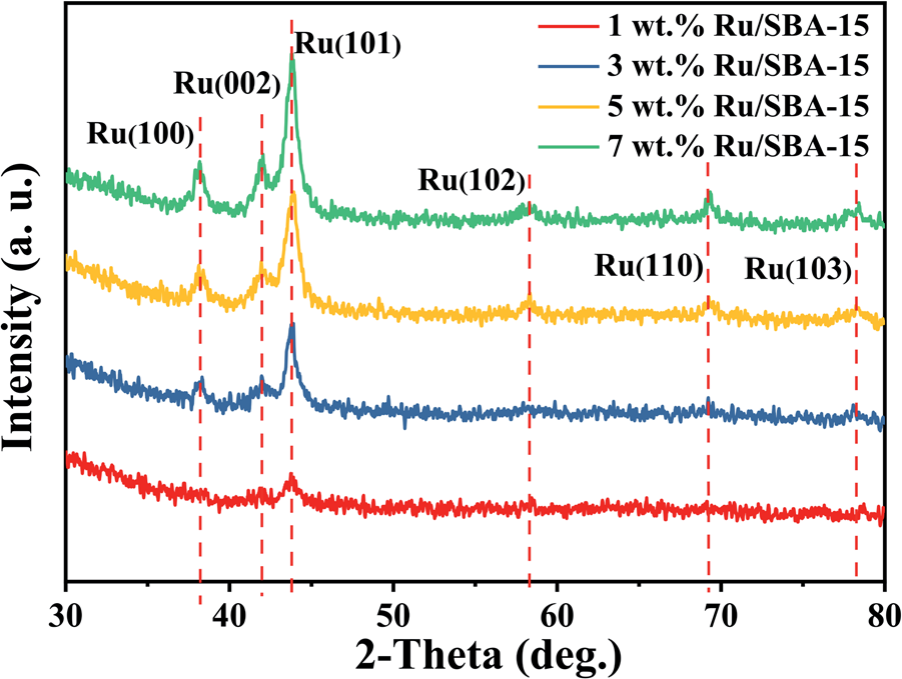
XRD patterns of the Ru/SBA-15 catalysts.

**Fig. 3:**
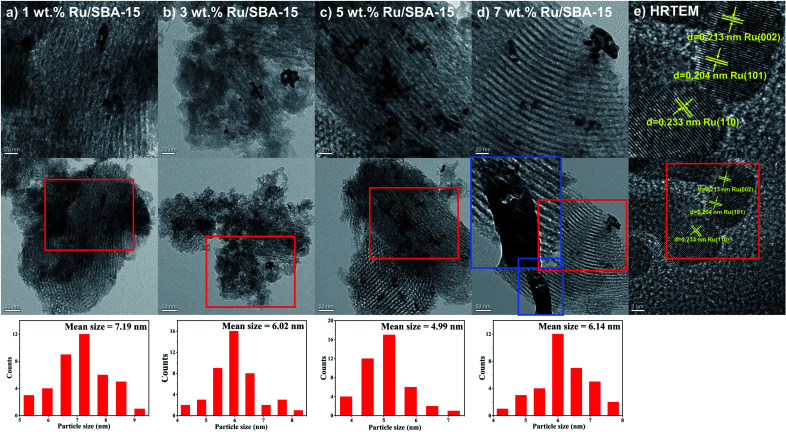
TEM images of (a–d) 1–7 wt% Ru/SBA-15 and (e) HRTEM image of 5 wt% Ru/SBA-15.

**Fig. 4:**
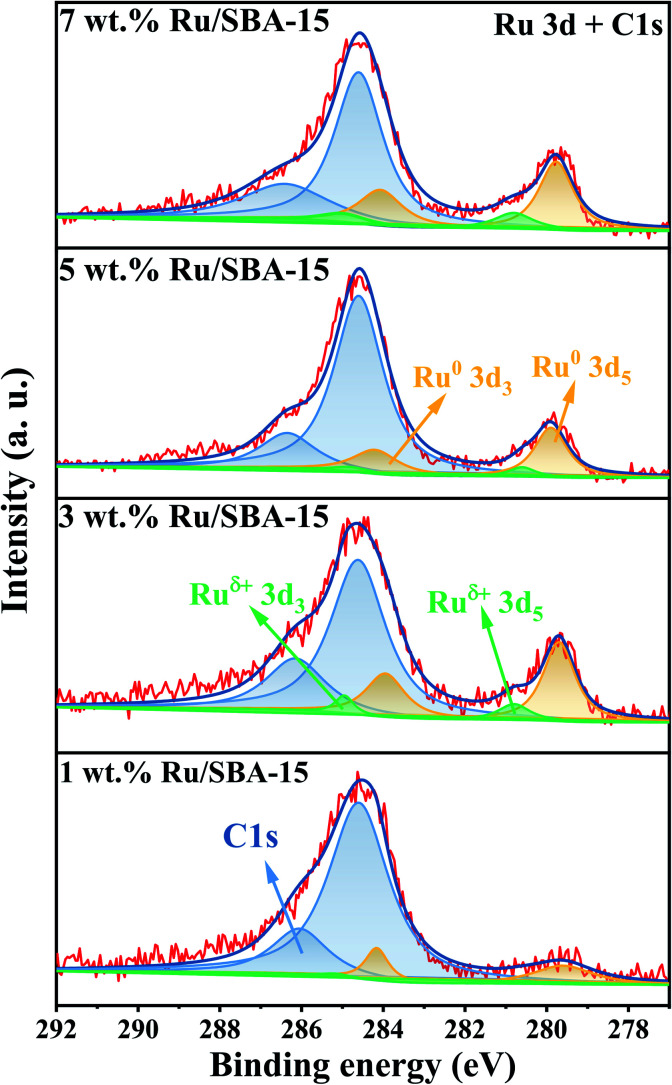
XPS spectra of Ru 3d and C 1s.

The chemical states of Ru with different loadings were evaluated by XPS. As illustrated in Fig. 4. A strong overlap between the Ru 3d and C 1s regions could be observed, and the two prominent peaks exhibited at 284.8 and 286.4 eV can be assigned to C 1s.^29,30^ The metal species can be divided into Ru^0^ (279.9 and 284.1 eV) and Ru^δ+^ (280.2 and 284.4 eV), and the specific binding energies of Ru 3d were given in ESI (Table S3)† along with a quantitative estimation of the surface elements. 5 wt% Ru/SBA-15 exhibited a high content of surface Ru^0^, which accounted for 87.0% of surface Ru species. It was speculated that Ru^0^ was the active phase of the catalyst and Ru^*δ*+^ played a role of stabilizing the catalyst.^29^

### 3.2 Phenol hydrogenation

Aqueous-phase hydrogenation of phenol with different Ru loadings (1 wt% to 7 wt%) on SBA-15 was performed. It was found that the hydrogenation of phenol enhanced to the best over 5 wt% Ru/SBA-15 at 80 °C for 1.5 h with a selectivity of 91.8% to cyclohexanol, which was higher than those on other Ru loadings ones (Table 2, entries 1–4), and the variation tendency was identical to that of Ru dispersion. According to N_2_ adsorption–desorption, the pore diameter of all the catalyst samples was about 2–9 nm. Thus, the phenol molecule could enter the catalyst channel and contact the active site unhinderedly with the molecular diameter of about 0.6 nm.^31^ The different activity of the catalysts might be originated from the amount and/or the crystal planes of Ru exposed. It was reported that the hydrogenation of phenol in aqueous solution favorably occurred on the Ru edge site, and the hydrogenation of phenol was kinetically favored on the Ru (101) lattice face with the low-coordination sites of Ru particles.^32^ Hence, the high dispersive Ru particles with clear edges, and the Ru (101) found as the main exposed crystal plane from TEM and XRD results might be responsible for the highest catalytic activity of 5 wt% Ru/SBA-15 with the turnover number (TON) of 172.84 based on the conversion of reactant within 1.5 h. Therefore, 5 wt% Ru/SBA-15 catalyst was selected for further investigation.

**Table 2:** Hydrogenation of phenol under different conditions^a^

Entry	Catalyst (Ru loading wt%)	*T* (°C)	*t* (h)	Conv. (%)	Sel.^b^ (%)	
					C–OH	C=O
1	1	80	1.5	33.0	51.9	48.1
2	3	80	1.5	55.8	59.3	40.7
3	5	80	1.5	86.2	91.8	8.2
4	7	80	1.5	63.5	68.2	31.8
5	5	20	5	>99.9	>99.9	<0.1
6	5	40	4	>99.9	>99.9	<0.1
7	5	60	3.5	>99.9	>99.9	<0.1
8	5	80	3	>99.9	>99.9	<0.1
9	5	100	3	>99.9	>99.9	<0.1

a Reaction conditions: phenol (5 mmol), Ru/SBA-15 (0.2 g), H_2_O (50 mL), *P*_H_2__ = 2 MPa. With >99.9% conversion and selectivity, the minimum time required for reaction completion is given.

b Cyclohexanone and cyclohexanol were the only reaction products observed.

The effects of reaction time and temperatures were conducted. Phenol will be converted to cyclohexanone and cyclohexanol over 5 wt% Ru/SBA-15 even at room temperature. The time needed to reach the complete conversion of phenol to cyclohexanol at different temperature were listed in Table 2 (entries 5–9). At a low temperature of 20 °C, the conversion of >99.9% was achieved with a selectivity to cyclohexanol exceeding 99.9% when the reaction time was prolonged to 5 h, indicating the high performance under mild conditions comparing with literature (Table S1†). When the temperature increased to 80 °C, the time to reach phenol complete conversion was reduced to 3 h. However, the time to reach the complete conversion was not significantly reduced by further increasing the reaction temperature to 100 °C, and there was almost no variation of selectivity. The highly selective transformation of phenol to cyclohexanol at room temperature requires no external heating, allowing for the derivation of very practical production routes.

**Fig. 5:**
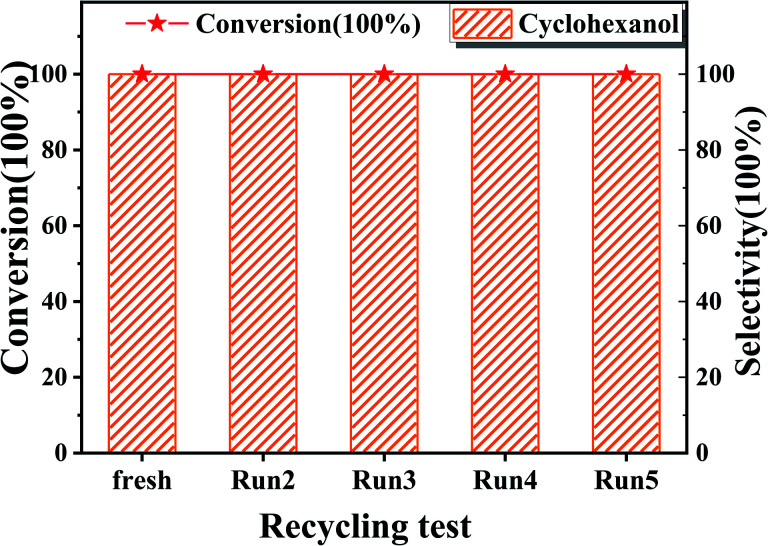
The recycling test of phenol on 5 wt% Ru/SBA-15 catalyst. Reaction conditions: phenol (5 mmol), 5 wt% Ru/SBA-15 (0.2 g), H_2_O (50 mL), *P*_H2_ = 2 MPa, 3 h.

The recyclability of Ru/SBA-15 catalyst at 80 °C was tested in five catalytic runs. Before the next catalytic reaction, the used Ru/SBA-15 catalyst was washed with EtOH and dried at 80 °C for 1 h without other treatments. It was shown in Fig. 5 that the conversion of phenol and selectivity to cyclohexanol all remained at >99.9% without loss of catalytic ability after 5 runs. Evidenced by XRD, XPS, TG and TEM data of the used Ru/SBA-15 catalyst shown in Fig. S1,† there might be no coke formed on 5 wt% Ru/SBA-15 after 5 runs according to the TG data, and there was no obvious agglomeration of Ru particles with the mean size of 4.93 nm. Moreover, the amount of Ru^0^ species on the surface of 5 wt% Ru/SBA-15 after 1 and 5 run was stabilized as about 85% (Table S4†), and the Ru(101) was also the major lattice face exposed after 5 run. So, the high stability of the catalyst could be ascribed to the stable Ru^0^ content and Ru(101) lattice face exposed, which was the main active species enhancing the hydrogenation of phenol.^20,21^

### 3.3 Kinetic studies of phenol hydrogenation with 5 wt% Ru/SBA-15

**Fig. 6:**
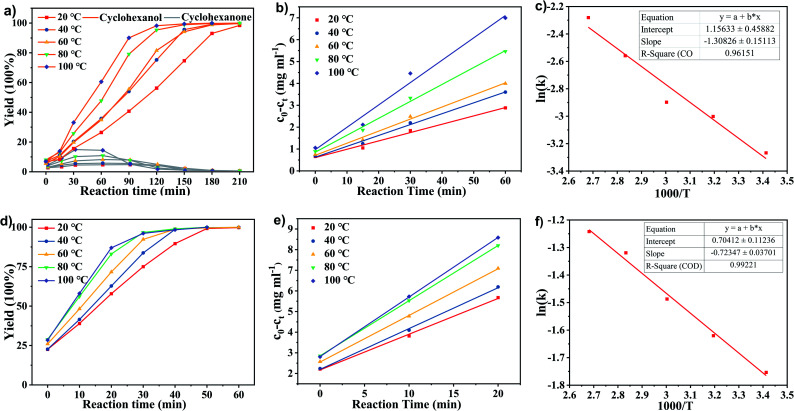
The yield of (a) cyclohexanol and cyclohexanone from phenol and (d) cyclohexanol from cyclohexanone, kinetics curves of the hydrogenation of (b) phenol and (e) cyclohexanone at different temperature with time on stream, and temperature dependence of the rate parameter in the Arrhenius plots for the conversion of (c) phenol and (f) cyclohexanone over 5 wt% Ru/SBA-15. Reaction conditions: phenol or cyclohexanone (5 mmol), 5 wt% Ru/SBA-15 (0.2 g), H_2_O (50 mL), *P*_H2_ = 2 MPa.

The kinetic studies of phenol hydrogenation over Ru/SBA-15 at different temperatures were carried out by measurement of the reaction rates at the initial stage. A temperature range of 20–100 °C was selected to determine the conversion of reactants with time on stream, and the corresponding results were summarized in Fig. 6. As illustrated in Fig. 6a, the reaction progressed with an increasing of cyclohexanol selectivity and a volcanic trend to the selectivity of cyclohexanone. Therefore, the hydrogenation of phenol to cyclohexanone and cyclohexanol proceeds in a sequential manner over the Ru/SBA-15 catalyst.^35–37^ The kinetic curves of phenol hydrogenation in initial stage (0–1 h, herein we definite the point heating from room temperature (∼10 °C) up to setting temperature as 0 h (about 15 min)) and associated Arrhenius plots were presented in Fig. 6b and c, respectively.

As shown in Fig. 6b, when plotting the substrate concentration consumed against the reaction time ((*c*_0_ − *c*_*t*_) − *t*, where *c*_0_ and *c*_*t*_ were the initial concentration of phenol and that at a reaction of time *t*, respectively), a straight line was obtained, and the rate constant *k* could be obtained from the slope of the line. The reaction rate of the phenol hydrogenation conformed to an equation of (*c*_0_ − *c*_*t*_) = *kt*, where the reaction rates of phenol hydrogenation at the initial stage over Ru/SBA-15 catalyst had nothing to do with the substrate concentration, but only depended on the reaction temperature with the corresponding results shown in Table 3. It was speculated that the adsorbed reactant was saturated on the surface of the catalyst. In this case, the concentration of phenol was saturated relative to the active site, so, the hydrogenation reaction on the Ru/SBA-15 has zero-order character for the concentration of phenol. Generally, it was considered that the apparent activation energy (Ea) can be approximated as constant under kinetically controlled conditions applied with the standard methodologies.^38,39^ Hence, the apparent activation energy (Ea) and the pre-exponential factor (*A*) could be obtained by the associated Arrhenius plots of ln (*k*)−1000/*T* shown in Fig. 6c, where a very low Ea of 10.88 kJ mol^−1^ and A of 3.18 mg mL^−1^ min^−1^ explained the high catalytic activity of the hydrogenation of phenol over Ru/SBA-15, which can proceed smoothly and efficiently under mild conditions.

**Table 3:** The kinetic parameters for the hydrogenation of phenol in an aqueous phase

Entry	*T* (K)	*k*_1_ (×10^−2^ mg mL^−1^ min^−1^)	*R* ^2^	Ea_1_^a^ (kJ mol^−1^)	*A*_1_ a (mg mL^−1^ min^−1^)	*k*_2_ (×10^−2^ mg mL^−1^ min^−1^)	*R* ^2^	Ea_2_^a^ (kJ mol^−1^)	*A*_2_ a (mg mL^−1^ min^−1^)
1	293	3.81	0.986	10.88	3.18	17.32	0.996	7.38	2.02
2	313	4.97	0.992			19.79	0.998		
3	333	5.52	0.992			22.61	0.999		
4	353	7.74	0.984			26.74	0.999		
5	373	10.22	0.975			28.88	0.999		

a Ln *k* = −Ea/*RT* + ln *A*.

Herein, cyclohexanone was also used as raw materials to explore the reaction mechanism of phenol hydrogenation to cyclohexanol, for cyclohexanone was the only intermediate observed in the reaction. The reaction kinetics of cyclohexanone hydrogenation to cyclohexanol was investigated. As illustrated in Fig. 6d, a conversion of >99.9% from cyclohexanone to cyclohexanol could be achieved in 50 min at 20 °C, which implied that the hydrogenation of cyclohexanone to cyclohexanol was faster than that of phenol. There was also a uniform conversion rate at the initial stage of the reaction with a zero-rate character. Fig. 6e and f plotted the substrate concentration consumed against the reaction time ((*c*_0_ − *c*_t_) − *t*) and the associated Arrhenius plots, respectively, and the corresponding results of rate constant *k* were shown in Table 3. Currently, the rate constant of cyclohexanone hydrogenation was about 4.5 times higher than that of phenol hydrogenation at 20 °C. However, when the temperature increased to 100 °C, it was just about 2.8 times higher. The results indicated a relatively lower activation energy of cyclohexanone hydrogenation, for the rate constant of the reaction with higher activation energy increased more significantly with increasing the temperature. The activation energy (Ea) of cyclohexanone hydrogenation over the Ru/SBA-15 catalyst obtained by the slope of ln(*k*)-1000/T plotting (Fig. 6f) was 7.38 kJ mol^−1^, and the corresponding pre-exponential factor (*A*) was 2.02 mg mL^−1^ min^−1^. The lower Ea of cyclohexanone hydrogenation explained the higher selectivity of phenol to cyclohexanol, and was possibly associated with the behavior of adsorbates on active sides.^18,40^

**Scheme 1:**
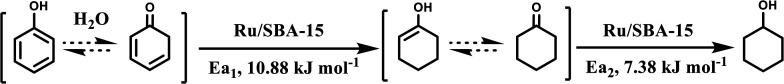
Proposed reaction pathway for the hydrogenation of phenol.

Combining the reaction rate constants of phenol and cyclohexanone hydrogenation over Ru/SBA-15 at different temperatures and the apparent activation energy obtained, the proposed reaction mechanism of phenol hydrogenation over Ru/SBA-15 catalyst was shown in Scheme 1. Phenol would form its keto isomer in water and firstly hydrogenated to cyclohexanone (Ea_1_ = 10.88 kJ mol^−1^), then cyclohexanone would be further hydrogenated to cyclohexanol (Ea_2_ = 7.38 kJ mol^−1^).^24,37,41^ Compared with the results reported for phenol hydrogenation in aqueous phase, Ru/SBA-15 gave a lower apparent activation energy in both of the two progress.^42,43^ The zero-order character of phenol hydrogenation suggested the weak adsorption and fast desorption of the molecules reacted on Ru/SBA-15 catalyst, which explained high activity of hydrogenation and stability of the recycling reactions without carbon deposition on the catalyst.

### 3.4 Hydrogenation and demethoxylation of methoxy-substituted phenols

Given the great hydrogenation activity of Ru/SBA-15 catalyst for phenol, the hydrogenation reaction was further studied with typical lignin-derived phenols as substrates so as to explore the hydrogenation and demethoxylation activity of the catalyst. The results were shown in Table 4. It could be seen that the conversion rate of H-type lignin was over 99.9%, and the products were all corresponding alkyl-substituted cyclohexanols without residual ketone intermediates, Table 4 (entries 1–3). When using G- and S-type monophenols as the substrate, the conversion also reached >99.9% as shown in Table 4 (entries 4–9). The selectivity of demethoxylation product (cyclohexanol) and hydrogenation product (2-methoxycyclohexanol) of guaiacol were 25.2% and 74.8%, respectively (entry 4). Remarkably, the presence of *para*-alkyl group of C_aryl_–OH improved the yield of demethoxylation product to 33.2% and 33.6%, respectively (entries 5–6), and the *para*-alkenyl group improved the yield of cyclohexanol further to 37.4% (entry 7). The temperature (80 °C) of demethoxylation here was significantly lower compared with those in literature (Table S1†). As for syringol, the selectivity to cyclohexanol, 2-methoxycyclohexanol and 2,6-dimethoxycyclohexanol was 10.8%, 60.9% and 28.3%, respectively (entry 8), and the selectivity to the corresponding products from 4-methylsyringol was similar to syringol (entry 9).

**Table 4:** Hydrogenation of lignin-derived phenols over 5 wt% Ru/SBA-15^a^

Entry	Substrate	Conv. (100%)	Products and selectivity (100%)
1	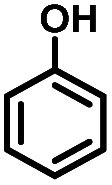	>99.9	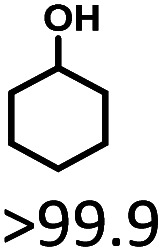
2	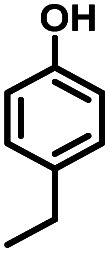	>99.9	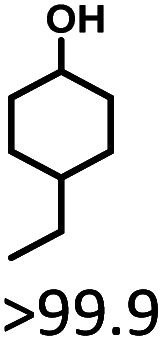
3	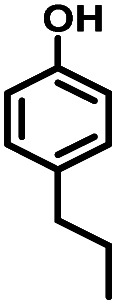	>99.9	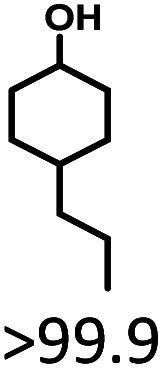
4	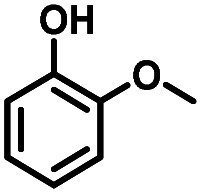	>99.9	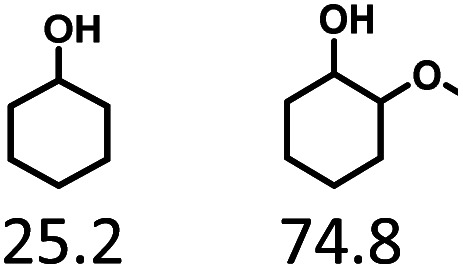
5	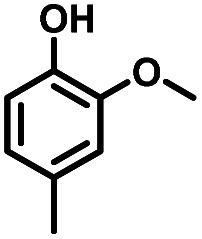	>99.9	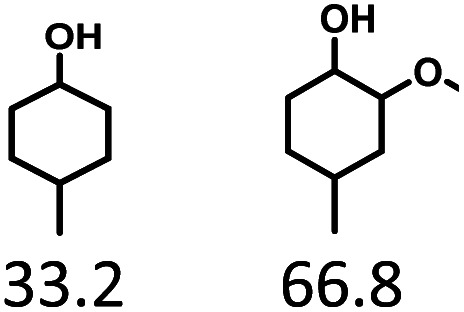
6	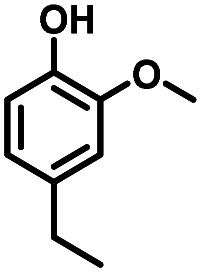	>99.9	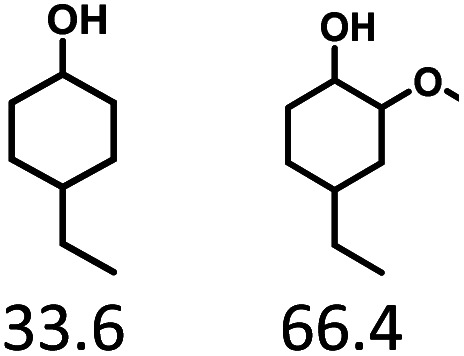
7	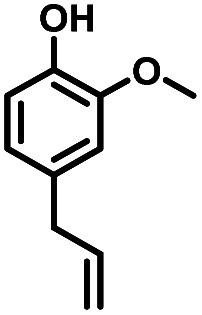	>99.9	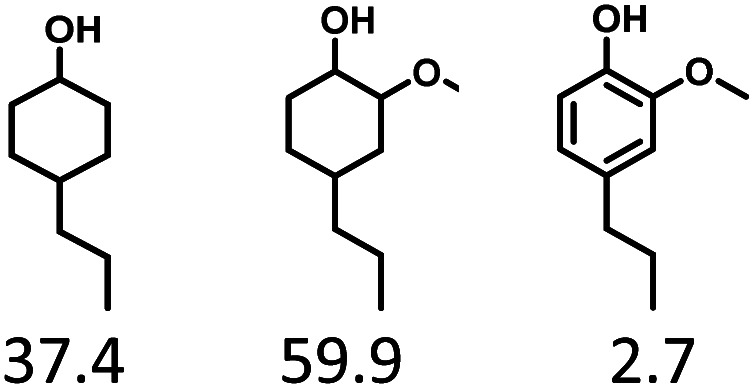
8	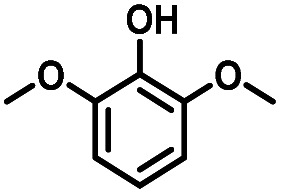	>99.9	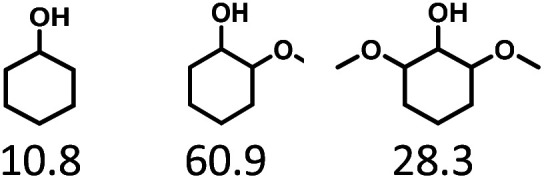
9	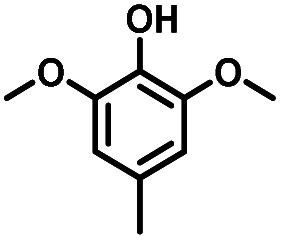	>99.9	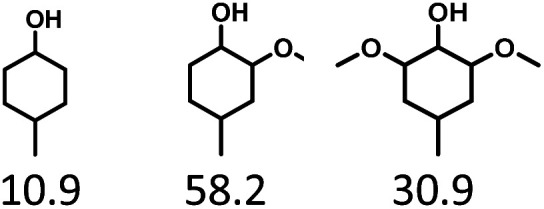

a Reaction conditions: lignin-derived phenols (5 mmol), 5 wt% Ru/SBA-15 (0.2 g), H_2_O (50 mL), *P*_H_2__ = 2 MPa, 4 h.

**Fig. 7:**
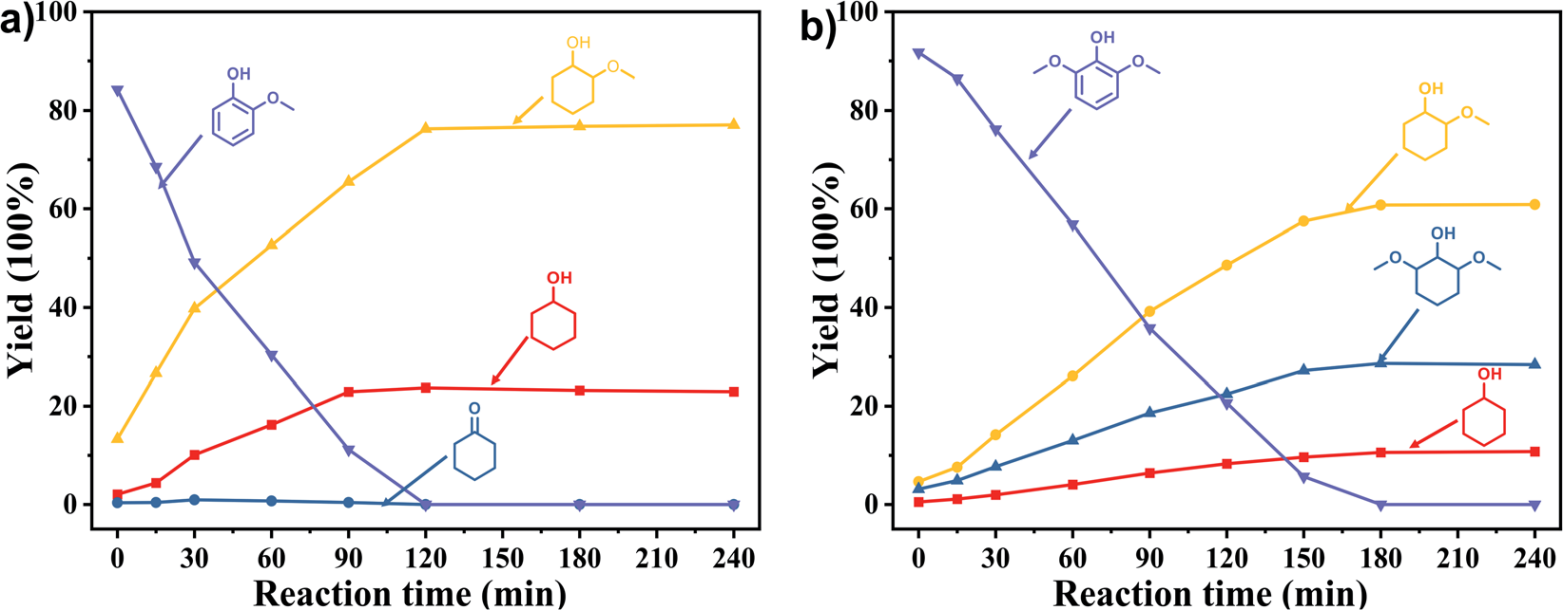
Product distribution with the extension of time of (a) guaiacol and (b) syringyl as substrates. Reaction conditions: substrate (5 mmol), 5 wt% Ru/SBA-15 (0.2 g), H_2_O (50mL), *P*_H2_ = 2 MPa.

In order to reveal the catalytic pathway of the demethoxylation and hydrogenation of methoxy-substituted monophenols, reactions with the extension of time were carried out to examine the product distribution as a function of time (Fig. 7). As shown in Fig. 7a for the HDO of guaiacol, there was a small amount of cyclohexanone existed and maintained at a relatively low level with the yield reached about 1.2% at 0.5 h. By prolonging the reaction time, the yield of cyclohexanone decreased gradually, which was speculated to be converted to cyclohexanol. The yield of cyclohexanol and 2-methoxycyclohexanol increased gradually with the final ratio of about 1 : 3 (25.2%:74.8%), and there was no trend of methoxycyclohexanol transformation to cyclohexanol. Hence, it was speculated that the hydrogenation and demethoxylation processes occurred simultaneously and competitively with the ratio of about 4 : 1. Fig. 7b showed the product distribution of syringol with the extension of reaction time, and the demethoxylation rate was relatively increased compared with that of guaiacol, where 10.8% cyclohexanol was obtained with the loss of two methoxy groups, and the yield of 2-methoxycyclohexanol (60.9%) was higher compared with 2,6-dimethoxycyclohexanol (28.3%). Therefore, the hydrogenation and demethoxylation rate might be 10 : 8. This result explained the phenomenon of the better demethoxylation effects of mixed monophenols than single substrate, which was possibly due to different adsorption behavior of the substrate on the catalyst and the steric hindrance effect of methoxy groups.^3,44^ Remarkably, there was no phenol or guaiacol detected even at 0 h as the reaction has just risen to the set temperature for the HDO of guaiacol and syringol, which can be attributed to the excellent catalytic activity of Ru species in the hydrogenation of aromatic ring.^20,21^

**Scheme 2:**
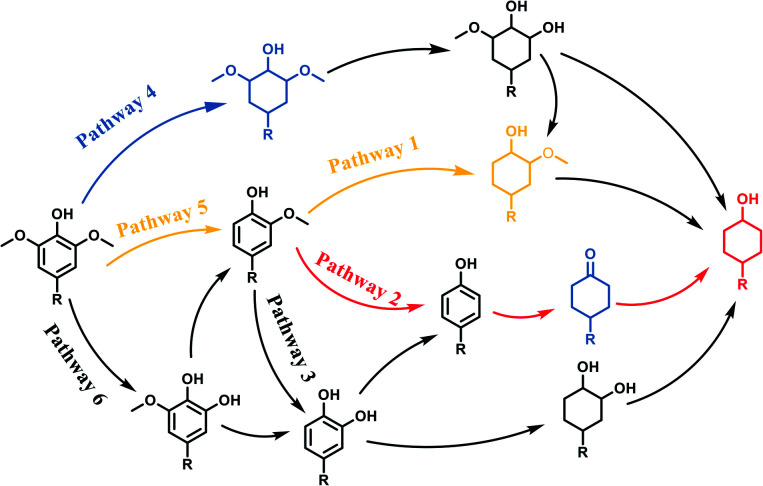
Reaction Pathways for lignin-derived phenols hydrogenate to cyclohexanols.

Scheme 2 showed the pathways of monophenols conversion to cyclohexanols. It could be speculated that there were mainly three pathways for guaiacol conversion:^20,44–46^ the saturation hydrogenation of aromatic ring to obtain 2-methoxycyclohexanol firstly and then the demethoxylation (pathway 1); the demethoxylation (C_aryl_–OCH_3_ cleavage) to obtain phenol firstly and then the hydrogenation of aromatic ring (pathway 2); and the demethylation (C_aryl_O–CH_3_ cleavage) to obtain diphenols firstly and then the hydrogenolysis of C_aryl_–OH group or the hydrogenation of aromatic ring (pathway 3). As for syringol, there were also three main pathways for the HDO: the saturation hydrogenation of aromatic ring to obtain 2,6-dimethoxycyclohexanol followed by the loss of methoxy groups (pathway 4), the demethoxylation to guaiacol and then pathway 1 to obtain 2-methoxycyclohexanol or pathway 2 to obtain cyclohexanol (pathway 5); the demethylation first upon the hydrogenation or demethoxylation (pathway 6). Obviously, Ru/SBA-15 catalyst exhibited a higher activity of aromatic hydrogenation compared with deoxygenation, which might be resulted from the fact that the electron-donating C_aryl_–OH group can stabilize the transition state carbocation then decrease the demethoxylation rate.^45,46^ The deoxygenation order of cracking the related bonds might be C_aryl_–OCH_3_ > C_alkyl_–OCH_3_ > C_aryl_–OH according to the distribution of the products. Due to the fact that the C_aryl_–OH group can form a conjugated structure with the aromatic ring, the activation energy of the C_aryl_–OH bond was the highest, and there always needs a higher temperature of more than 200 °C to get complete deoxidation products of cyclohexane derivatives from phenols.^47–50^ In addition, the absence of Brønsted acid sites on SBA-15 will lead to the inactivity for the dehydration of cyclohexanol and promote the hydrogenation of monophenols to stay in alcohols.^51^ The cleavage of C_aryl_–OCH_3_ was easier than C_aryl_O–CH_3_ for the phenyl was a better electron attraction group compared with methyl for the C_aryl_–O–CH_3_ ether bonds,^24^ and the adsorption on Ru species could lower the activation energies of the C_aryl_–OCH_3_.^15^ Similarly, C_alkyl_–OCH_3_ was more difficult to cleavage compared with C_aryl_–OCH_3_ for the steric hindrance and electronic effect will restrain the cleavage of C_alkyl_–OCH_3_ bond and result in a higher temperature to achieve this process.^52,53^ However, there were no C_aryl_O–CH_3_ or C_alkyl_–OCH_3_ cleavage intermidates detected, thus it could not speculate the cracking order of the two bonds over Ru/SBA-15 under the conditions investigated.

Therefore, increasing demethoxylation activity or reducing hydrogenation activity were reliable approaches to increase the yield of cyclohexanol.^46,54^ There has been reports of increasing temperature or changing the structure of catalyst to increase demethoxylation activity.^13,20,55,56^ However, starting from the structure of the reactants, the presence of *para*-alkyl group increased the yield of cyclohexanol under mild conditions, which might be due to the fact that the presence of *para*-alkyl group broke the conjugate environment and decreased the bond energy of C_aryl_–OCH_3_, then the C_aryl_–OCH_3_ bond could be cleaved easier under mild conditions. In contrast, the unsaturated *para*-alkenyl group might reduce the hydrogenation activity of aromatic ring for there was residual non-cyclic hydrogenation products (propyl guaiacol, 2.7%) of allyl guaiacol, and the yield of cyclohexanol further increased to 37.4%. The addition of methoxyl group might also increase the competitiveness of demethoxylation based on the distribution of the products of syringol. To verify this assumption, a mixture of phenol, 4-ethyl phenol, guaiacol, 4-ethyl guaiacol, 4-allyl guaiacol, and syringol was used to simulate lignin depolymerization liquid products and evaluate the catalytic performance of Ru/SBA-15, and the specific yields were shown in Table 5. The results showed that all the substrates were hydrogenated completely, and the increased amounts of cyclohexanol and 4-ethylcyclohexanol indicated the increased demethoxylation effect in the HDO of the mixture. In addition, the methoxy-substituted monophenols were demethoxylated even better than using only one phenol compound as reactant, which indicated the applicability of Ru/SBA-15 catalyst to lignin derived phenols.

**Table 5:** The yield of hydrogenation of lignin-derived liquid mixture^a^

Substracts (mg mmol^−1^)	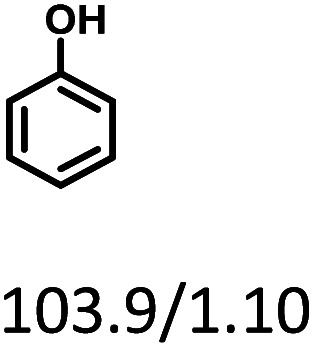	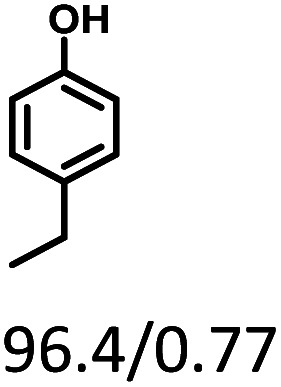	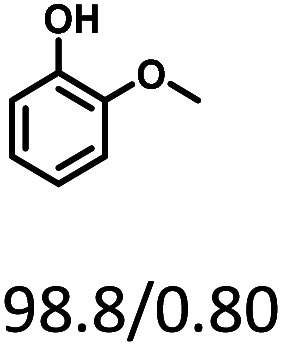	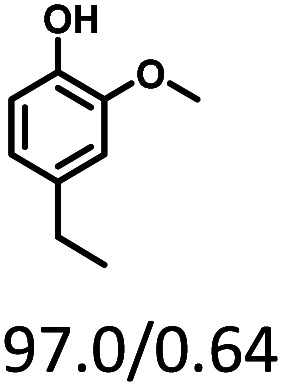	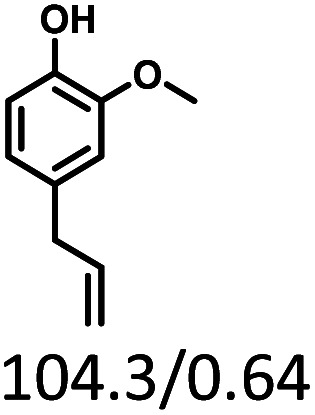	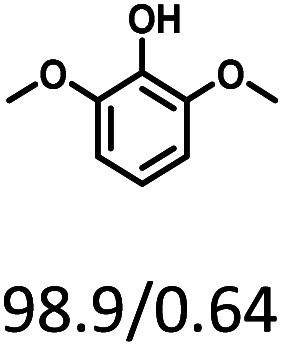	
Products (mg mmol^−1^)	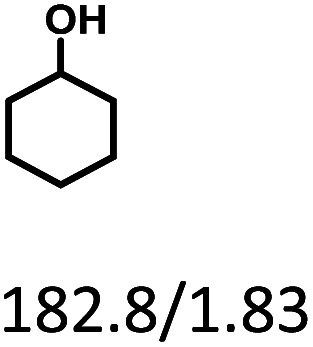	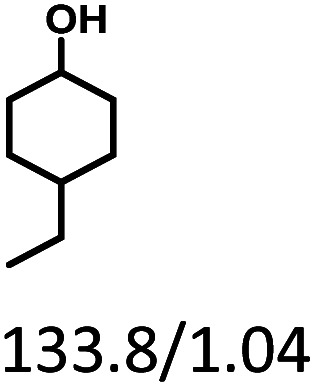	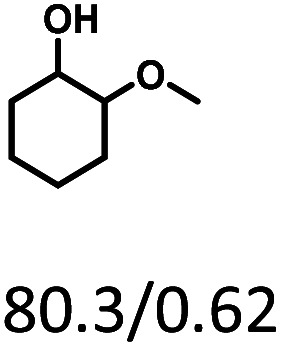	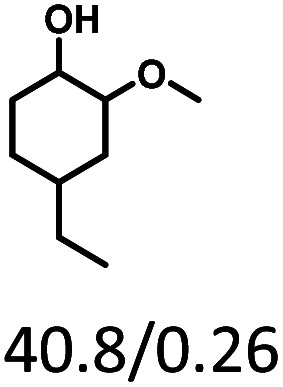	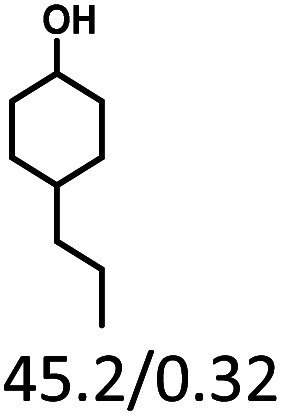	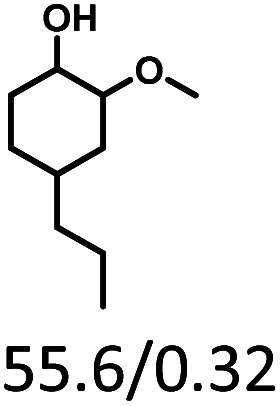	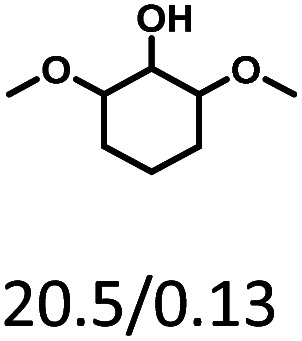

a Reaction conditions: lignin-derived monophenols (0.6 g), 5 wt% Ru/SBA-15 (0.2 g), H_2_O (50mL), *P*_H_2__ = 2 MPa, 4 h.

## 4. Conclusions

Ru/SBA-15 showed an excellent hydrogenation activity of monophenols to cyclohexanol. The yield of cyclohexanol from phenol hydrogenation was >99.9% in an aqueous system at 20 °C for 5 h. The hydrogenation reaction of phenol catalyzed by Ru/SBA-15 conforms to the zero-order character and the apparent activation energy of the reaction was 10.88 kJ mol^−1^, which indicated the high and stable catalytic activity of hydrogenation. Simultaneously, Ru/SBA-15 showed competitively demethoxylation activity according to the product distribution from the HDO of G- and S-type monophenols, whereas the rate of hydrogenation was faster than demethoxylation. The complete hydrogenation product had no tendency of demethoxylation based on the variation of product distribution with reaction time, for the steric hindrance and electronic effect will restrain the cleavage of C_alkyl_–OCH_3_ bond, which needs a higher temperature. It was indicated that the Ru/SBA-15 catalyst might have good performance on the hydrodeoxygenation of lignin to alcohols under mild conditions according to the structure of lignin, where the existing of unsaturated branched chains and methoxyl groups would enhance the competitively of demethoxylation.

## Author contributions

Shanshan Feng: conceptualization, methodology, validation, formal analysis, investigation, data curation, writing – original draft, visualization. Xudong Liu: methodology, investigation. Zhishan Su: methodology, investigation. Guiying Li: methodology, investigation. Changwei Hu: supervision, project administration, funding acquisition, writing – review & editing.

## Conflicts of interest

The authors declare no competing financial interest.

## Supplementary Material

RA-012-D2RA01183B-s001
