# Supplementary material for: Low temperature catalytic hydrodeoxygenation of lignin-derived phenols to cyclohexanols over the Ru/SBA-15 catalyst
Source: RSC Adv. 2022 Mar 24;12(15):9352–62. doi: 10.1039/d2ra01183b (PMC8985087; doi:10.1039/d2ra01183b)
Supplement: RA-012-D2RA01183B-s001 [file RA-012-D2RA01183B-s001.pdf]

Electronic Supplementary Material (ESI) for RSC Advances.
This journal is © The Royal Society of Chemistry 2022

Electronic Supplementary Information

Low temperature catalytic Hydrogenation of Lignin-Derived Phenols to cyclohexanols over Ru/SBA-15 Catalyst

Shanshan Feng [a], Xudong Liu [b], Zhishan Su [a], Guiying Li [a], Changwei Hu [a]*

[a]Key Laboratory of Green Chemistry and Technology, Ministry of Education, College of Chemistry, Sichuan University, Chengdu, Sichuan 610064, P. R. China.

[b]State Key Laboratory of Utilization of Woody Oil Resource, Hunan Academy of Forestry Sciences, Changsha 410004, China

* Corresponding author: Changwei Hu (C. Hu)

Tel/Fax: +86-28-85411105

Email address: changweihu@scu.edu.cn

Experimental

Catalyst characterization

Transmission electron microscopy (TEM) and high-resolution transmission electron microscopy (HR-TEM) images were measured in a TECNAI G2-20 TWIN (FEI Co., German) instrument with an acceleration voltage of 200 kV to analyze the dispersity and crystal orientation of Ru species. The samples were dispersed in ethanol by ultrasonication and dropped onto copper grids for observation. The particle diameter (d) of Ru particles were counted by the software of Nano Measurer.

N_2 adsorption-desorption isotherms were collected with a Micromeritics Tristar II 3020 analyzer to analyze the surface areas and the pore volumes of the catalyst samples. The surface areas and pore volumes were determined by the Brunauer-Emmett-Teller (BET) Barrett-Joyner-Halenda (BJH) equation, respectively. Before analysis, the samples were heated at 120 ^{o}C and 300 ^{o}C for 2 h under vacuum, respectively.

Powder X-ray diffraction (XRD) were obtained using a Shimazu XRD-6100 diffractometer with Cu-Kα monochromatized radiation (λ =1.5406 Å) at 40 kV and 30 mA to analyze the crystalline phase of the catalysts. The scanning angle were recorded over the 2θ range of 5–80 ^{o} at a scan rate of 5 ^{o} min^{-1}.

X-ray photoelectron spectroscopy (XPS) measurements were carried out on an AXIS Ultra DLD (KRATOS) spectrometer with a monochromatic Al-Kα X-ray source to analyze the chemical state of supported metals. The binding energy was internally calibrated by setting the C1s peak at 284.8 eV.

Inductively coupled plasma atomic emission spectroscopy (ICP-AES) was

performed by a Thermo Elemental IRIS Advantage ER/S spectrometer (Thermo Elemental, MA, USA) to measure the actual metal loading of the catalysts. The samples were dissolved in aqua regia and HF, then diluted with deionized water before measurement.

Thermogravimetric analysis (TG) was used to identify the coke deposition on the used catalysts by a NETZSCH STA 449 F5 analyzer. About 0.01 g sample was placed in an alumina crucible with a platinum crucible cover. The sample was heated to 800 ^{o}C with 10 ^{o}C min^{-1} under 60 mL min^{-1} dry air flow.

Catalytic tests

The hydrogenation of lignin-derived phenols was performed in a 100 mL Parr reactor. In a typical reaction, 5 mmol substrate with 0.2 g of Ru/SBA-15 and 50 mL water were loaded in the reactor, which was flushed with N_2 for 3 times, and finally filled with 2 MPa H_2 for reaction. Then the reactor was set to desired temperature with stirring at 400 rotation per minute. The reaction was performed for scheduled time and then cooled down by fan for about 20 min to room temperature. After filtration, the fluid was analyzed qualitatively and quantitatively by GC-MS (Agilent 6820) and GC-FID (PERKINELMER Clarus 580) for yield calculation. A DB-5 capillary column (30 m ×0.25 mm×0.25 μm) was used both in GC and GC-MS, and the temperature program was set as rising with a ramp of 5°C min$^{−1}$ from 50 °C to 200 °C and maintaining for 5 min. For the GC-FID instrument, the flow rate of nitrogen was 1 mL min^{-1}, the injector temperature was 280 ^{o}C and the detector temperature was set at 330 ^{o}C mainly to ensure that the sample could be completely vaporized to avoid the contamination of the

detector. Acetophenone was used as internal standard.

Table S1 Hydrodeoxygenation (HDO) selectivity of lignin-derived phenols in literature and this work.

Entry	catalyst	reactant	solvent	Reaction conditions (T, t, P)			Conv. (%)	Selectivity [a]				Ref.
				T/°C	t/h	P/MPa		RCol	RCone	RCane	RPh	
1	Rh-H	phenol	H_2O	30	6	5	100	100				1
2	Pd/γ-Al_2O_3	phenol	H_2O	60	12	2	100	100				2
3	Pd–HAP	phenol	H_2O	75	3	1	100		100			3
4	Pd@mpg-C_3N_4	phenol	H_2O	45	12	0.1	99		98.5			4
5	Pd/C+$AlCl_3$	phenol	CH_2Cl_2	30	12	1	>99.9		99.9			5
6	Ru/5Nb_2O_5-5MC	phenol	H_2O/decalin	200	4	1	100			100		6
7	Ru/SBA-15	phenol	H_2O	20	5	2	>99.9	100				This study
8[b]	Ru/C	G	acetic acid	80	4	3	24	25				7
9	RuZrLa-2	G	H_2O	160	4	4	100	81				8
10	Ru–MnOx/C	G	H_2O	160	2	1.5	99.9	79				9
11[c]	NiRu-HT	AG	isopropanol	150	6	2.5	100	83.8				10
12[d]	Ni/SiO_2-Al_2O_3	PG	hexadecane	250	0.5	1	100	85				11
13	Ni/Beta	G	-	300	-	4	100			76		12
14	Ru/HZSM-5-OM	AG	H_2O	170	4	4	>99.5			83		13
15	Au/TiO_2+ HZSM-5	PG	benzene	350	2	10	100				60	14
16	Ru/SBA-15	AG	H_2O	80	4	2	>99.9	37.4				This study

[a] 4-alkylcyclohexanol (RCol), 4-alkylcyclohexanone (RCone), 4-alkylcyclohexane (RCane), 4-alkylphenol (RPh); [b] guaicol (G); [c] 4-allylguaiacol (AG); [d] 4-propylguaicol (PG).

Table S2 Ru particle diameter (d_{XRD}) calculated by Ru(101) in XRD patterns.

Catalyst	β (FWHM)	2θ	d_{XRD} (nm)[a]
1 wt.% Ru/SBA-15	0.465	43.756	18.21
3 wt.% Ru/SBA-15	0.539	43.739	15.71
5 wt.% Ru/SBA-15	0.759	43.958	11.17
7wt.% Ru/SBA-15	0.607	43.838	13.96

[a] $d_{XRD}=K\lambda/(\beta\cos\theta)$, where K is constant (0.89), λ is the wavelength of X-rays (0.15406).

Table S3 Specific binding energies of Ru 3d of Ru/SBA-15 catalysts.

Sample	Name	Position (eV)	Area	FWHM (eV)	%Cont	Ru^0/ (Ru^0+ $Ru^{\delta+}$)
1 wt.% Ru/SBA-15	Ru^0 $3d_{5/2}$	279.60	37.55	1.75	54.7	91.2
	Ru^0 $3d_{3/2}$	284.16	25.04	0.67	36.5	
	$Ru^{\delta+}$ $3d_{5/2}$	280.60	3.63	1.20	5.3	
	$Ru^{\delta+}$ $3d_{3/2}$	285.17	2.42	0.75	3.5	
3 wt.% Ru/SBA-15	Ru^0 $3d_{5/2}$	279.70	91.14	0.94	50.3	83.9
	Ru^0 $3d_{3/2}$	283.95	60.76	1.13	33.5	
	$Ru^{\delta+}$ $3d_{5/2}$	280.80	17.55	0.90	9.7	
	$Ru^{\delta+}$ $3d_{3/2}$	284.95	11.70	0.53	6.5	
5 wt.% Ru/SBA-15	Ru^0 $3d_{5/2}$	279.90	97.01	0.95	52.2	87.0
	Ru^0 $3d_{3/2}$	284.20	64.67	1.31	34.8	
	$Ru^{\delta+}$ $3d_{5/2}$	280.60	14.50	0.75	7.8	
	$Ru^{\delta+}$ $3d_{3/2}$	284.80	9.67	1.03	5.2	
7 wt.% Ru/SBA-15	Ru^0 $3d_{5/2}$	279.80	119.64	1.00	50.1	83.6
	Ru^0 $3d_{3/2}$	284.07	79.76	1.25	33.4	
	$Ru^{\delta+}$ $3d_{5/2}$	280.81	23.50	1.00	9.9	
	$Ru^{\delta+}$ $3d_{3/2}$	284.99	15.68	1.20	6.6	

Table S4 Specific binding energies of Ru 3d of used Ru/SBA-15 catalysts.

Sample	Name	Position (eV)	Area	FWHM (eV)	%Cont	$Ru^0/(Ru^0+Ru^{\delta+})$
5 wt.% Ru/SBA-15-run 1	Ru^0 $3d_{5/2}$	280.11	515.59	1.20	51.0	84.9
	Ru^0 $3d_{3/2}$	284.39	343.73	1.40	34.0	
	$Ru^{\delta+}$ $3d_{5/2}$	280.60	91.42	1.30	9.0	
	$Ru^{\delta+}$ $3d_{3/2}$	284.88	60.95	1.98	6.0	
5 wt.% Ru/SBA-15-run 5	Ru^0 $3d_{5/2}$	279.85	295.25	0.78	51.1	85.2
	Ru^0 $3d_{3/2}$	284.09	196.83	0.86	34.1	
	$Ru^{\delta+}$ $3d_{5/2}$	280.50	69.35	1.04	12.0	
	$Ru^{\delta+}$ $3d_{3/2}$	284.85	16.23	1.07	2.8	

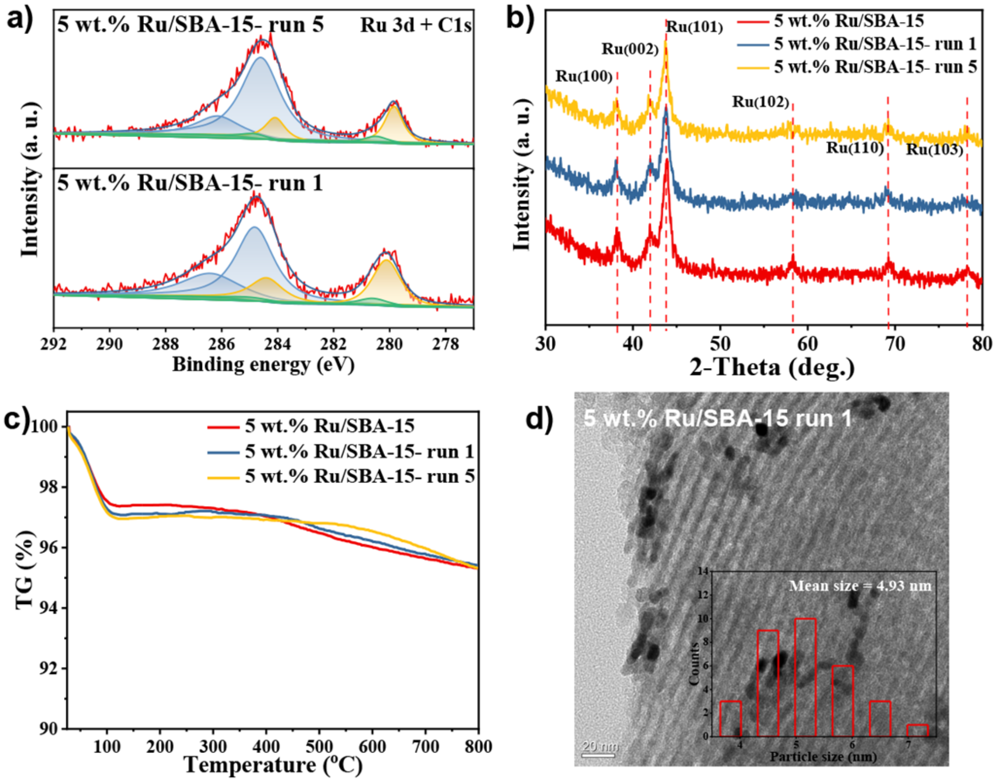

Figure S1 a) XRD[a], b) XPS[b], c) TG[c] and d) TEM[d] data of the used Ru/SBA-15 catalysts.

[a] The specific binding energy of used 5 wt.% Ru/SBA-15 showed in Table S2 indicated that the Ru0 content (~85%) of the used catalysts had no obvious decrease compared with the fresh catalyst (87.0%).

[b] The characteristic diffraction peaks of Ru^0 observed in XRD patterns of the used catalysts was the same with the fresh sample.

[c] The weight loss at about 100 °C might be attributed to water, and there was no other form of weightlessness both in the fresh and used catalysts.

[d] The particle diameter of the used 5 wt.% Ru/SBA-15 catalyst showed no increase compared with the fresh sample with the mean size of 4.93 nm, and there was no obvious agglomeration, which could account for the high activity and stability of the catalyst in the aqueous system.

Reference

1. L. Chen, A. P. van Muyden, X. Cui, G. Laurenczy and P. J. Dyson, *Green Chem.*, 2020, **22**, 3069-3073.
2. J. Yi, Y. Luo, T. He, Z. Jiang, J. Li and C. Hu, *Catalysts*, 2016, **6**.
3. G. Xu, J. Guo, Y. Zhang, Y. Fu, J. Chen, L. Ma and Q. Guo, *ChemCatChem*, 2015, **7**, 2485-2492.
4. Y. Wang, J. Yao, H. Li, D. Su and M. Antonietti, *J. Am. Chem. Soc.*, 2011, **133**, 2362-2365.
5. T. J. Huizhen Liu, * Buxing Han,* Shuguang Liang, Yinxi Zhou, *science*, 2009, **326**, 1250-1252.
6. C. Zhang, C. Jia, Y. Cao, Y. Yao, S. Xie, S. Zhang and H. Lin, *Green Chem.*, 2019, **21**, 1668-1679.
7. C. E. J. J. Vriamont, T. Chen, C. Romain, P. Corbett, P. Manageracharath, J. Peet, C. M. Conifer, J. P. Hallett and G. J. P. Britovsek, *ACS Catal.*, 2019, **9**, 2345-2354.
8. G.-Y. Xu, J.-H. Guo, Y.-C. Qu, Y. Zhang, Y. Fu and Q.-X. Guo, *Green Chem.*, 2016, **18**, 5510-5517.
9. M. Ishikawa, M. Tamura, Y. Nakagawa and K. Tomishige, *Appl. Catal., B*, 2016, **182**, 193-203.
10. A. Sreenavya, A. Sahu and A. Sakthivel, *Ind. Eng. Chem. Res.*, 2020, **59**, 11979-11990.
11. W. Schutyser, G. Van den Bossche, A. Raaffels, S. Van den Bosch, S.-F. Koelewijn, T. Renders and B. F. Sels, *ACS Sustainable Chem. Eng.*, 2016, **4**, 5336-5346.
12. P. Yan, M. M.-J. Li, E. Kennedy, A. Adesina, G. Zhao, A. Setiawan and M. Stockenhuber, *Catal. Sci. Technol.*, 2020, **10**, 810-825.
13. L. Wang, J. Zhang, X. Yi, A. Zheng, F. Deng, C. Chen, Y. Ji, F. Liu, X. Meng and F.-S. Xiao, *ACS Catal.*, 2015, **5**, 2727-2734.
14. X. Huang, J. M. Ludenhoff, M. Dirks, X. Ouyang, M. D. Boot and E. J. M. Hensen, *ACS Catal.*, 2018, **8**, 11184-11190.
